# Gene-Level Analyses of Novel Olfactory-Related Signal from Severe SARS-CoV-2 GWAS Reveal Association with Disease Mortality

**DOI:** 10.3390/covid5120206

**Published:** 2025-12-14

**Authors:** Yu Chen Zhao, Xinan Wang, Yujia Lu, Rounak Dey, Yuchen Liu, Francesca Giacona, Elizabeth A. Abe, Emma White, Li Su, Qingyi Wei, Xihong Lin, Lorelei A. Mucci, Jehan Alladina, David C. Christiani

**Affiliations:** 1 Department of Epidemiology, Harvard T.H. Chan School of Public Health, Boston, MA 02115, USA; 2 Department of Environmental Health, Harvard T.H. Chan School of Public Health, Boston, MA 02115, USA; 3 Department of Biostatistics, Harvard T.H. Chan School of Public Health, Boston, MA 02115, USA; 4 Pulmonary and Critical Care Division, Department of Medicine, Massachusetts General Hospital, Harvard Medical School, Boston, MA 02115, USA; 5 Department of Medicine, Duke University School of Medicine, Durham, NC 27710, USA; 6 Department of Population Health Sciences, Duke University School of Medicine, Durham, NC 27710, USA

**Keywords:** SARS-CoV-2, genetics, respiratory disease

## Abstract

**Importance:**

The coronavirus disease 2019 (COVID-19) was the third leading cause of mortality in the United States for three years in a row. The genetic contributions to disease severity remain unclear and many previously identified single nucleotide polymorphisms (SNPs) have not been replicated nor linked with functional significance.

**Objective:**

To identify SNPs associated with mortality among hospitalized COVID-19 patients supplemented by expression quantitative trait loci (eQTL) evidence to infer plausible functional mechanisms related to COVID-19 severity.

**Design:**

A quality-controlled genome-wide association study (GWAS) supported by robust gene-level omnibus kernel association tests (SKAT-O), functional prediction, and eQTL analyses of the top GWAS signal.

**Setting:**

Massachusetts General Hospital (MGH).

**Participants:**

370 adult ICU patients with SARS-CoV-2 infection and acute hypoxemic respiratory failure and floor patients with mild hypoxemia managed with supplemental oxygen consecutively admitted to MGH between March and June 2020 (Surge 1), and January and March 2021 (Surge 2) with baseline clinical characteristics and demographics collected.

**Exposures:**

Low-pass genotyped SNPs from whole blood and aggregated SNP-sets of potential disease susceptibility loci with ±500 kb flanking regions.

**Main Outcomes & Measures:**

Genome-wide individual SNP associations and SNP-set associations with mortality outcomes from 370 severe COVID-19 cases.

**Results:**

After LD pruning (<0.8) and false discovery rate adjustment (<0.05), we identified rs7420371 G>A of the receptor transporter protein 5 (*RTP5*) gene as the top independent signal significantly associated with 30- and 60-day mortality among severe COVID-19 patients (OR, 2.32; 95% CI, 1.59–3.39; *p* = 4.92 × 10^−9^ and OR, 2.06; 95% CI, 1.43–2.97; *p* = 5.43 × 10^−8^, respectively). SKAT-O analyses on the *RTP5* SNP-set showed associations with both mortality outcomes (*p* = 5.90 × 10^−5^ and 6.17 × 10^−5^, respectively). eQTL analysis showed rs7420371 A allele significantly upregulated the mRNA expression of *RTP5* in 266 cerebellum tissues, in 277 cerebellar hemisphere tissues, and in 270 cerebral cortex samples.

**Conclusions & Relevance:**

We discovered a novel, independent, and potentially functional SNP *RTP5* rs7420371 G>A to be significantly associated with COVID-19 mortality. The A allele is significantly associated with elevated mRNA expression of *RTP5* in the brain, an important protein coding gene that modulates olfactory binding and taste perceptions in response to SARS-CoV-2 infection.

## 1. Introduction

The coronavirus disease 2019 (COVID-19), a disease caused by the severe acute respiratory syndrome coronavirus 2 (SARS-CoV-2), evolved rapidly into a global pandemic [1,2]. As of July 2023, more than 103 million cases and 1.1 million deaths were reported due to COVID-19 in the United States (US), making it the third leading cause of mortality in the US for three-years in a row [3,4]. Patients infected with SARS-CoV-2 exhibit varied clinical manifestations, ranging from asymptomatic to mild to severe diseases [4]. While vaccination status and age have been shown to heavily influence disease presentation and clinical outcome, studies have found variations in the disease severity within fully vaccinated and unvaccinated individuals, which suggests that there may be other factors contributing to the disease severity [4,5].

To date, genome-wide associations studies (GWASs) on COVID-19 have identified many single nucleotide polymorphisms (SNPs) and corresponding disease susceptibility loci (DSL) that are associated with disease severity and progression, which may explain some of the observed differences in disease presentation [4,5]. However, despite efforts directed towards the discovery of the molecular determinants behind the varied clinical manifestations of COVID-19, the pathophysiological consequences of these genetic variants remain unclear. Many of the identified SNPs have not been replicated in follow-up studies, nor are these SNPs linked with known functional annotations, which limits the interpretability and clinical application of these findings [4–6]. Hence, the discovery of novel genome-wide signals and the validation of previously identified genetic variants are critical to advance our understanding of the role of genetic variants in determining COVID-19 severity [6,7]. In the present study, we aim to (i) identify SNPs that are associated with 30- and 60-day mortality among a cohort of hospitalized COVID-19 patients; (ii) validate previously identified genetic signals by utilizing summary statistics from the COVID-19 Host Genetics Initiative (HGI) with powerful optimally unified (omnibus) statistical models [7,8]; and (iii) perform functional prediction and expression quantitative trait loci (eQTL) analyses in normal tissues on the identified SNPs to infer plausible pathophysiological mechanisms that may contribute to COVID-19 severity.

## 2. Materials & Methods

### 2.1. Study Population

The present study included a cohort of 370 severe COVID-19 patients, where disease severity is defined as hospitalization due to SARS-CoV-2 infection, including both medical floor and intensive care unit (ICU) admissions. We enrolled adult ICU patients with SARS-CoV-2 infection and acute hypoxemic respiratory failure (AHRF) and floor (non-ICU) patients with mild hypoxemia managed with 2–6 L/min supplemental oxygen who were admitted consecutively to the Massachusetts General Hospital (MGH) between March and June 2020 (Surge 1), and between January and March 2021 (Surge 2) in Boston, Massachusetts. We collected baseline clinical characteristics including smoking status and body mass index (BMI), patient demographics (e.g., age at hospitalization, sex, race), and 30- and 60-day mortality outcomes from the electronic medical records (EMR). Whole blood DNA samples were collected from excess clinical blood draws in EDTA-treated tubes and genotyped using low-pass whole genome sequencing (LPWGS) on the Gencove platform.

The institutional review board of the Massachusetts General Hospital had approved of the present study (Protocol #2015P001650), and informed consent was waived permitting use of the previously collected data from clinical records.

### 2.2. Patients and Public Involvement

Patients and the public were not directly involved in the design or conduct of this research. This study utilized data and samples from patients who were enrolled during their hospitalization for COVID-19 at Massachusetts General Hospital between March–June 2020 and January–March 2021. While patients were enrolled for sample collection during their hospital stay, this was done as part of routine clinical care, with excess blood samples being used for research purposes. The research questions, study design, and outcome measures were developed by the research team based on clinical considerations without direct patient input. Patients were not involved in the recruitment procedures beyond providing samples as part of their clinical care, nor in the interpretation or dissemination of results.

### 2.3. Genotype Quality Control

The genotyping data of the 370 severe COVID-19 patients were imputed using the Genotype Likelihoods Imputation and Phasing Method (GLIMPSE) [9] and there were 80,650,091 SNPs available for downstream analyses. For quality control (QC), we assessed and controlled for sex discrepancy, removed SNPs where the proportion of missing SNPs per individual > 0.01, removed SNPs on the X chromosome and those with minor allele frequency (MAF) < 0.05, and removed SNPs with a Hardy–Weinberg Equilibrium (HWE) *p*-value < 1 × 10^−5^. No participants were removed while assessing for potential cryptic relatedness by thresholding proportion identity by descent (IBD) to 0.2. After all QC protocols, 6,305,694 SNPs remained as candidate common SNPs for further analyses. Principal Component Analysis (PCA) was performed to compare the underlying genetic ancestry to electronic health record-documented race and ethnicity (Supplementary Figure S3).

### 2.4. Genome-Wide Association Analysis

We performed a multivariable logistic regression to test the association between individual SNPs and all-cause mortality at 30 and 60 days, adjusting for age, sex, smoking status, ICU status, and COVID-19 surge waves as precision predictors, and adjustment for potential population stratification using the top 10 principal components (PCs). The decision to adjust for a higher number of PCs was determined by inspecting the distribution of eigenvectors that showed substantial admixture from the Hispanic population in our study. Odds ratios (OR) and 95% confidence intervals (CI) were calculated as measures of association modeling each SNP under the additive genetic model. Multiple testing correction was conducted using the Bonferroni correction at alpha = 0.05, equivalent to the 5 × 10^−8^ genome-wide significance threshold assuming one million independent SNPs. The Benjamini–Hochberg False Discovery Rate (FDR) has been calculated to provide information on SNPs discovered under looser discovery threshold but was not used as the determinant for statistical significance in the present study. We utilized Manhattan plots and regional association plots to visualize the locations and linkage disequilibrium (LD) of our top signals. Quantile-quantile (QQ) plots and the genomic inflation factor lambda (λ_GC_) were used to evaluate potential inflation and to assess whether the adjustment for the top 10 PCs accounted for potential confounding that may be induced by potential population stratification. The top signal based on the Bonferroni-adjusted *p*-value and LD pruning was selected for further analysis. We then performed in silico replication attempts using the most closely related public data from release 7 of the COVID-19 HGI [7].

### 2.5. Optimally Adjusted SNP-Set Kernel Association Test

The omnibus/optimally adjusted SKAT model (SKAT-O) was used for all subsequent gene-level statistical analysis based on its ability to retain statistical power by aggregating multiple SNP effects within a gene, that is robust to the number of true signals or the direction and magnitude or their effects, which are often not known *a priori* [8]. We performed association tests with SKAT-O to validate and independently evaluate the gene-level effect of the top signal from our GWAS while additionally exploring the association between previously identified top genetic signals for hospitalization and COVID-19 mortality from the COVID-19 HGI [7]. The list of SNPs within genetic regions corresponding to the top signals from COVID-19 HGI were retrieved using the BiomaRt R package (v2.54.1) under the Genome Reference Consortium Human Build 37 (GRCh37) and matched with our post-QC SNP list [10,11]. We then calculated the genotype matrix for each respective gene and generated the null SKAT-O model required for the association analysis. Since the present study focused exclusively on common genetic variants, we specified the CommonRare parameter accordingly. Given the SKAT-O assumptions for sample size and the small sample size of our severe COVID-19 GWAS (*n* = 370), we applied the small sample adjustment for all samples to account for the increased uncertainty and variability that accompany small sample GWASs under the SKAT-O model and adjusted for ancestry PCs and demographic variables in the analysis [8].

All data and statistical analyses were conducted using PLINK v1.9, R v4.2.1, and SAS v9.4, unless otherwise indicated [12–14].

### 2.6. Functional Prediction & eQTL

To examine further the statistically significant and potentially functional SNPs obtained from our severe COVID GWAS, we performed an eQTL analysis leveraging the genomic data from the 1000 Genomes Project and the Genotype-tissue Expression (GTEx) Portal using 266 cerebellum tissue samples, 277 cerebellar hemisphere tissue samples, 270 cerebral cortex tissue samples, 601 lung tissue samples, and 800 whole blood samples [15,16]. We then performed functional prediction using publicly available bioinformatics tools, including RegulomeDB v2.0.3, HaploReg, and the ENCODE project [17–19].

## 3. Results

### 3.1. Genome-Wide Significance

**Figure 1.**
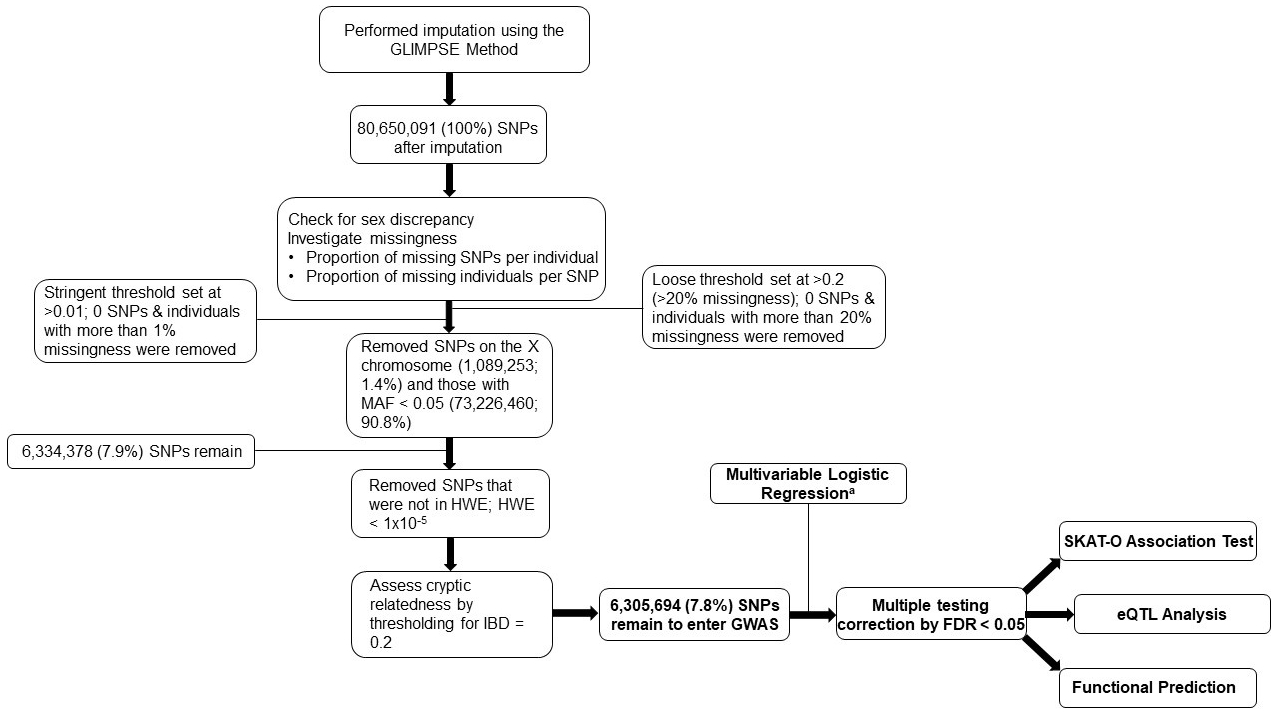
Study Design and Quality Control Flowchart. The study design and quality control procedures conducted in the present study. This is a discovery-only study using an original cohort of 370 hospitalized patients from the Massachusetts General Hospital. ^a^Adjusted for age, sex, smoking status, ICU status, COVID-19 surge waves, and for population stratification using the top 10 PCs. Abbreviations: GLIMPSE, genotype likelihoods imputation and phasing method; SNPs, single nucleotide polymorphisms; MAF, minor allele frequency; HWE, Hardy-Weinberg Equilibrium; IBD, identity by descent; GWAS, genome-wide association study; FDR, false discovery rate; SKAT-O, optimally adjusted sequence kernel association test; eQTL, expression quantitative trait loci; PC, principal components.

Basic demographics and clinical characteristics of the 370 patients with severe COVID-19 are described in Table 1. Notably, nearly 77% of all patients were over age 50, 57% of all patients were never smokers, and over 77% of all patients were overweight or obese (BMI > 25). The overall study design workflow and the genotype quality control methodologies of the present study are shown in Figure 1. After quality control, 6,305,694 SNPs remained as candidate SNPs for further analyses. After multiple testing correction by the Bonferroni correction ≤ 0.05, we identified 12 SNPs that were significantly associated with 30-day mortality and 4 SNPs that were significantly associated with 60-day mortality. All significant SNPs were located on the chr2: 241869729-241873823 locus. We then performed LD pruning (LD < 0.8) among the SNPs that were statistically significant for both 30- and 60-day mortality among severe COVID-19 patients to select independent SNPs. The rs7420371 G>A SNP of the receptor/chemosensory transporter protein 5 (*RTP5*) gene was determined as the top signal after considering LD among the SNPs and both the significance of the multivariable adjusted genome-wide *p*-values (*p* = 4.92 × 10^−9^ for 30-day mortality; *p* = 5.43 × 10^−8^ for 60-day mortality) and the Bonferroni-adjusted alpha values (0.031 for 30-day mortality; 0.050 for 60-day mortality) (Supplementary Figure S1A,B) [20].

Multivariable logistic regression with adjustment for age, sex, smoking status, ICU status, and COVID-19 surge waves showed that the *RTP5* rs7420371 A allele was significantly associated with a higher 30- (OR = 2.32; 95% CI: 1.59, 3.39) and 60-day mortality (OR = 2.06; 95% CI:1.43, 2.97), compared with the G allele under the additive genetic model (Table 2).

We then performed in silico replication attempts using the most closely related public data from release 7 of the COVID-19 HGI across two similar phenotypes to our comparison. Specifically, we attempted the replication in two large cohorts, the 23andme A2 cohort comparing very severe respiratory confirmed COVID-19 vs. population controls and the 23andme B1 cohort comparing hospitalized vs. non-hospitalized COVID-19 patients [7]. In both replication analyses, *RTP5* rs7420371G>A did not show a statistically significant association with the risk of severe COVID-19 (OR = 0.991, SE = 0.0137, *p*-value = 0.5199) or risk of hospitalization (OR = 0.986, SE = 0.0211, *p*-value = 0.5203). The full results from the replication are included in Supplementary Table S3.

### 3.2. SNP-Set (Sequence) Kernel Association Test

We applied the optimally adjusted SKAT-O model to validate the top genome-wide signal *RTP5* rs7420371 G>A by assessing the association between the *RTP5* SNP-set and mortality outcomes. The *RTP5* SNP-set with ±500 kb flanking regions were retrieved and matched with the post-QC severe COVID-19 GWAS SNP list (Supplementary Table S1). The *RTP5* SNP-set was significantly associated with 30- and 60-day mortality, with adjustment for age, sex, smoking status, ICU status, COVID-19 surge waves, and the top 10 PCs (*p* = 5.90 × 10^−5^ and 6.17 × 10^−5^, respectively). To explore further and to contextualize the association between previously identified genetic signals for hospitalization from the COVID-19 HGI, we applied SKAT-O to test the association between the top eight genes from the COVID-19 HGI that are associated with COVID-19 hospitalization, compared with population controls (i.e., *THBS3*, *SFTPD*, *MUC5B*, *ELF5*, *FBRSL1*, *SLC22A31*, *NR1H2*, and *TMPRSS2*) and the SNPs that reside within ±500 kb of these genetic regions within our severe COVID-19 GWAS. The goal of this is to evaluate whether hospitalization-associated severity markers might also influence post-hospitalization mortality to explore potential shared biology across severity stages. From the HGI explorations, we found that the *THBS3* SNP-set was significantly associated with 30- and 60-day mortality, with adjustment for age, sex, smoking status, and COVID-19 surge waves (*p* = 0.001 and 0.0005, respectively). The *SFTPD* SNP-set was also significantly associated with 30- and 60-day mortality, with adjustment for the same covariates and the top 10 PCs (*p* = 2.04 × 10^−5^ and 3.85 × 10^−5^, respectively). The *MUC5B* SNP-set was also significantly associated with 30- and 60-day mortality, with adjustment for the same covariates and PCs (*p* = 1.42 × 10^−5^ and 3.50 × 10^−5^, respectively). The same statistical analysis protocol was applied to the remaining candidate genetic regions, and we found robust statistical associations between all eight SNP-sets of interest (i.e., *THBS3*, *SFTPD*, *MUC5B*, *ELF5*, *FBRSL1*, *SLC22A31*, *NR1H2*, and *TMPRSS2*) and 30- and 60-day mortality (Table 3). The total number of SNPs retrieved for each genetic region and the total number of SNPs matched with our post-QC GWAS are shown in Supplementary Table S1.

### 3.3. eQTL and Functional Prediction

In the RNA-sequencing data of tissue samples from European descendants available from the GTEx project, the *RTP5* rs7420371 A allele was found to be significantly associated with an increased expression level of *RTP5* in 266 cerebellum tissues (*p* = 1.77 × 10^−6^, Figure 2A), in 277 cerebellar hemisphere tissues (*p* = 0.0439, Figure 2B), and 270 cerebral cortex samples (*p* = 0.0183, Figure 2C) from the GTEx RNA-sequencing data [16]. In contrast, the *RTP5* rs7420371 A allele was not statistically significantly associated with a change in expression levels of *RTP5* in 604 lung tissues and 803 whole blood samples (*p* = 0.944 and 0.388, respectively, Figure 2D,E) [16]. The median expression analysis of *RTP5* across all body sites revealed substantially elevated reads per kilobase million (RPKM) in most regions of the brain, with the highest median expression found in the hypothalamus (31.87 RPKM) and the total median expression level at 129.07 RPKM (Supplementary Figure S2). Further inspections using the Functional Annotation of Variants Online Resource (FAVOR) database showed that the rs7420371 SNP had high protein functional scores and low error rate, with a PHRED score for aPC protein function of 27.67 (top 0.17 percentile of the genome) [21]. The polymorphisms phenotyping (PolyPhen) score of 0.985 (“probably damaging” classification) and the Sorting Intolerant From Tolerant (SIFT) score of <0.05 suggests that the mutation is likely to disrupt protein functions which may lead to altered phenotypes (Supplementary Table S2) [21]. Lastly, we performed functional prediction utilizing publicly available bioinformatics tools, including ENCODE, RegulomeDB, and HaploReg [17–19]. Based on functional predictions using RegulomeDB (score = 1 f), the rs7420371 G>A showed substantial evidence for eQTL, transcription factor binding, and deoxyribonuclease (DNase) peak, which suggests plausible functionalities in affecting protein binding and modification of the expression of its target gene *RTP5* (Supplementary Table S2) [17–19].

## 4. Discussion

In the present study, we identified a potentially functional and independent SNP (i.e., *RTP5* rs7420371 G>A) that was significantly associated with a higher 30- and 60-day mortality of the hospitalized COVID-19 patients.

*RTP5* is a protein coding gene located on the q-arm of chromosome 2 (2q37.3) that codes for the receptor/chemosensory transporter protein 5, which enables and modulates olfactory receptor binding activities and the sensory perception of the bitter taste [22,23]. The loss of these functions are common and well-established disease manifestations of COVID-19 [24]. The receptor/chemosensory transporter protein 5 belongs to the same family of accessory chaperones (RTP1-4) known to facilitate membrane targeting and functional expression of G-protein–coupled receptors (GPCR), especially olfactory receptors [22,23,25]. Although direct experimental data on *RTP5* are sparse, tissue expression databases suggest that *RTP5* is enriched in the brain and lymph nodes [16]. Since GPCR pathways are central to immune modulation and autonomic regulation [25], we consider it plausible, though speculative, that genetically driven differences in *RTP5* mRNA expression could influence neuro-immune or autonomic responses that contribute to severe COVID-19 mortality. From the Broad Institute Molecular Signature Database, we found that *RTP5* belonged to many key pathophysiological pathways and mechanisms that are intertwined with COVID-19 phenotypes, including the detection of chemical stimulus involved in the sensory perception of taste, protein localization to the cell membrane, protein insertion into the membrane, and olfactory receptor binding [26]. In the present study, we found that patients with the *RTP5* rs7420371 A allele, compared with the G allele, resulted in more than doubling of risk for 30- and 60-day mortality among severe COVID-19 patients under the additive genetic model. The MAF of rs7420371 is 0.2965 in the full cohort; the effect allele A frequency differed substantially between those who died (0.4935) and those who did not (0.25), potentially driving the strong association signal we observed in the odds ratio. This substantial difference in allele frequencies between mortality groups, rather than ancestry groups, gives a stronger support for a potentially true biological association. Further support from eQTL analyses using European samples from the GTEx project revealed the rs7420371 A allele to be significantly correlated with an increased mRNA expression level of *RTP5* in 266 cerebellum tissues, 277 cerebellar hemisphere tissues, and in 270 cerebral cortex samples [15]. To facilitate our understanding of the modulatory role played by the rs7420371 G>A in the mRNA expression levels of *RTP5*, we have conducted functional prediction and found that the rs7420371 is a missense variant with substantial eQTL evidence, which was echoed in the present study, as well as the potential for transcription factor binding and deoxyribonuclease (DNase) peak; these suggest plausible functionalities by the SNP in affecting protein binding and the mRNA expression of its target gene *RTP5* [17]. The observed expression level of *RTP5* was relatively low in the cerebellum and the cerebellar hemisphere, compared to other brain tissues (Supplementary Figure S2) [15]. Through expanded eQTL analysis in GTEx, several other brain tissues with higher *RTP5* expression were borderline but not statistically significant at the 0.05 level. However, despite the efforts in eQTL and functional predictions, the biological interpretation of these findings remain challenging. The observed eQTL effects may have several potential explanations. For instance, it could suggest that even small changes in expression in the cerebellar hemisphere and the cerebellum could have functional relevance or the eQTL effect might be capturing cell-type specific expressions that may be diluted in bulk tissue analysis.

No direct evidence from the current literature has linked *RTP5* (or SNPs within this locus) with COVID-19 transmission or disease severity, and no earlier studies have explored and interpreted the eQTL effects of the rs7420371 A allele on brain tissues. This is likely because most early COVID-19 GWASs focused primarily on mild or a combination of mild and severe cases. The definition of “severe” has also been heterogenous across studies, which varies by symptoms, physician judgement, ICU bed availability, and geographical locations [5,6]. For instance, medical floor admissions due to COVID-19 would be considered reasonably “severe” in the US, a country where healthcare is generally accessible, whereas this definition may change (and reasonably so) in another setting. Therefore, additional functional studies are warranted to validate the role of *RTP5* in affecting olfactory receptor binding, which could be related to the loss of smell and the malfunctioned sensory perception of taste commonly present among COVID-19 patients.

Among the top eight genes that were identified as genetic regions of interest from the COVID-19 HGI (i.e., *THBS3*, *SFTPD*, *MUC5B*, *ELF5*, *FBRSL1*, *SLC22A31*, *NR1H2*, and *TMPRSS2*), *SFTPD* that codes for surfactant protein D, *MUC5B* that codes for the Mucin 5B protein, and *ELF5* that codes for E74 Like ETS Transcription Factor 5 are the most novel and interesting genes that have strongly implications in COVID-19 pathogenesis and disease severity [27]. The *SFTPD* gene is part of the innate immune response that protects lungs against inhaled microbes and chemicals [27]; together with *MUC5B*, these two genes have been found to be upregulated among patients with chronic obstructive pulmonary disease (COPD) but mostly down regulated among severe COVID-19 patients [27–30]. *ELF5* was identified as a potential risk gene for severe COVID-19, which may be partially explained by the functional involvement of *ELF5* in the regulation and differentiation of epithelial cells of the lung [27,31].

We acknowledge some limitations in our study. Most importantly, despite the replication attempts, our study is a discovery-only GWAS. This design was necessitated by the data available at the time of the study. We acknowledge that the discovery-only approach may be susceptible to “winner’s curse” and may limit the interpretation and application of the primary GWAS findings reported in the present study. We were also not able to adjust for major comorbidities in our analysis, such as cardiovascular disease or hypertension, due to lacking the corresponding data, which could potentially confound the observed mortality risk. The 370 severe COVID-19 patients analyzed were mostly of European descent; thus, our findings may not be generalizable to other ethnic populations. The relatively small sample size of 370 severe COVID-19 patients, considered modest for GWAS, may limit the statistical power for discovery, which may have resulted in the inability to detect the weaker effects of some otherwise potentially important SNPs. The power limitation was partially mitigated by the implementation of the SKAT-O analysis, which served as support and indirect validation for our GWAS findings [8]. However, despite this effort, we acknowledge that our SNP-set analysis would be much more powerful in a larger population where multiple rare variants can demonstrate their impact [8]. While we adjusted for population stratification using the top 10 genetic principal components, the potential for residual confounding remains, particularly in cohorts with substantial admixture or cryptic relatedness. Further, the present study enrolled participants through COVID-19 surge waves in early 2020 and 2021, where the dominant SARS-CoV-2 subtypes and the proportion of vaccinated individuals differed considerably. Since the data on SARS-CoV-2 subtypes and vaccination were not available for the present analysis, we were unable to assess the impact when combining these effects. The top signal from our discovery GWAS was not statistically significant in both replication attempts using release 7 of the COVID-19 HGI data. We attempted replication of our top finding in the most recent COVID-19 HGI release, querying both the A2 (very severe respiratory COVID-19 vs. population) and B1 (hospitalized vs. non-hospitalized COVID-19) phenotypes. In both analyses, *RTP5* rs7420371 G>A did not show a statistically significant association. However, we want to emphasize that neither HGI phenotype directly matches our outcome of mortality among hospitalized COVID-19 patients; therefore, lack of replication in these datasets may not fully exclude a valid association in our specific clinical context. Despite substantial efforts in searching for a publicly available severe COVID-19 cohort with similar characteristics as our discovery cohort, we were unable to find a phenotypically and characteristically similar cohort that was publicly available. We believe our in silico replication attempts represent the closest feasible analysis with current resources, and we encourage future efforts to harmonize and publicly share summary statistics for mortality among hospitalized patients. Our study only distinguished ethnicity between Hispanic and non-Hispanic White patients. While we adjusted for genetic ancestry using PCs, residual confounding cannot be fully excluded. Using linear mixed models such as REGENIE may better capture the subtle population structure. Future studies in larger and mortality-specific cohorts are needed to validate these findings. Choosing SKAT-O as a method for common variant analysis also comes with its own benefits and limitations. Notably, SKAT-O is designed as a test for rare genetic variants; however, by specifying the CommonRare function to shift the weights between common and rare variants, SKAT-O is perfectly capable of analyzing common variant SNP-sets with slightly reduced (but overall improved) statistical power [8]. Given these limitations, we suggest that our findings be considered as preliminary and hypothesis-generating. The results should be interpreted with caution due to the modest sample size and a lack of statistically significant replication.

In summary, we performed a GWAS on a cohort of severe COVID-19 patients and discovered a novel, independent, and potentially functional SNP *RTP5* rs7420371 G>A to be significantly associated with the mortality outcomes of severe COVID-19 patients. We showed that patients with the A allele had significantly worse mortality outcomes compared with patients having the referent G allele. These results were supported by the powerful SKAT-O model and the eQTL analyses showed that the A allele of the rs7420371 SNP is potentially functional in the modulation of the mRNA expression levels of *RTP5*, an important protein coding gene involved in monitoring and modulating olfactory binding and the sensory perceptions of the bitter taste in response to SARS-CoV-2 infection. Despite the observed genome-wide associations between rs7420371 and severe COVID-19 mortality with support by powerful statistical models and in silico functional evidence, the molecular mechanisms of rs7420371 that underlies the observed associations remain ambiguous. Further functional validations that aim to unravel the role of the identified rs7420371 G>A SNP and the role of the SNPs within the *RTP5* region are warranted to enhance our understanding of the relationship between genetic variants, olfactory/taste malfunction, and COVID-19 severity.

## Supplementary Material

Table___Supplementary_Data

The following supporting information can be downloaded at: https://www.mdpi.com/article/10.3390/covid5120206/s1, Figure S1: Manhattan Plot for 30- and 60-Day Mortality; Figure S2: RTP5 Expression Levels from GTEx; Figure S3: Principal Component Analysis by Genetic Ancestry vs Electronic Health Records; Table S1: Total Number of SNPs Retrieved and Matched for each Candidate Genetic Region; Table S2: Functional Prediction of RTP5 rs7420371 G>A using RegulomeDB and FAVOR; Table S3: Replication Attempts using Public Data from Release 7 of the COVID-19 Host Genetics Initiative.

## Figures and Tables

**Figure 2.1:**
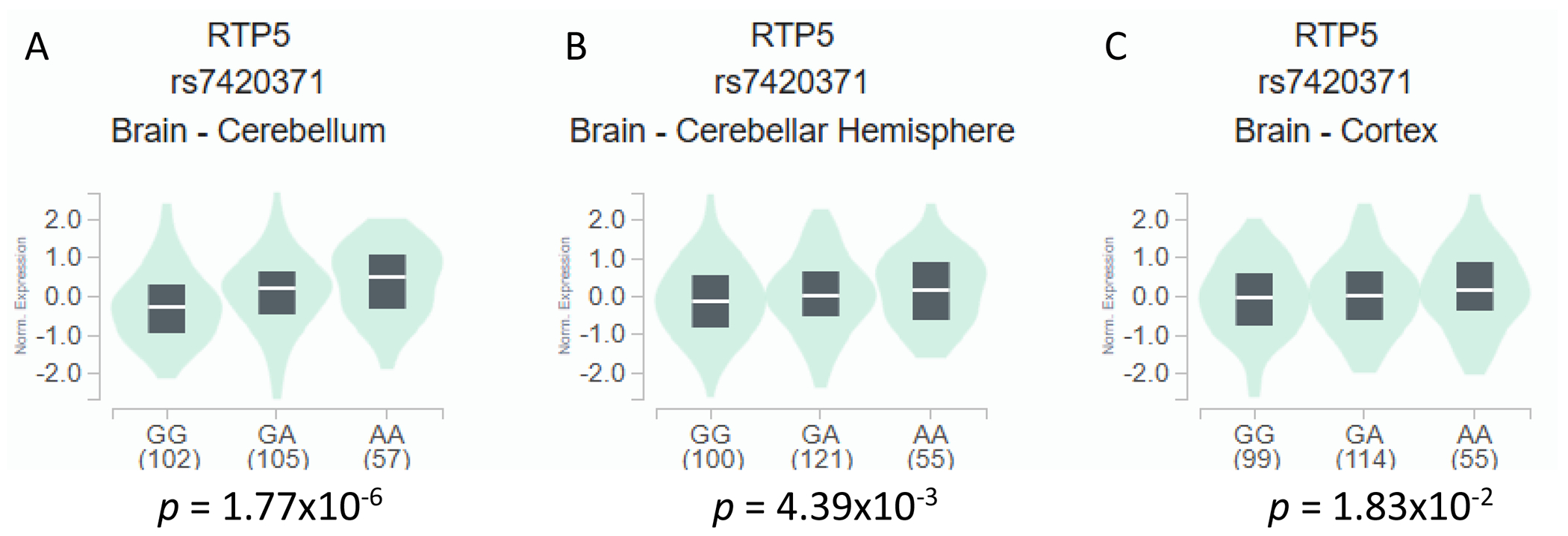
eQTL Analysis of rs7420371 G>A and mRNA Expression Levels of *RTP5* in the Cerebellum, Cerebellar Hemisphere, and the Cerebral Cortex. The rs7420371 A allele is significantly associated with an increased expression of *RTP5* in 266 brain cerebellum tissues from the GTEx database (A). The rs7420371 A allele is significantly associated with an increased expression of *RTP5* in 277 brain cerebellar hemisphere tissues from the GTEx database (B). The rs7420371 A allele is significantly associated with an increased expression of *RTP5* in 270 cerebral cortex tissues from the GTEx database (C). Abbreviations: eQTL, expression quantitative trait loci; mRNA, messenger RNA; RTP5, receptor/chemosensory transporter protein 5.

**Figure 2.2:**
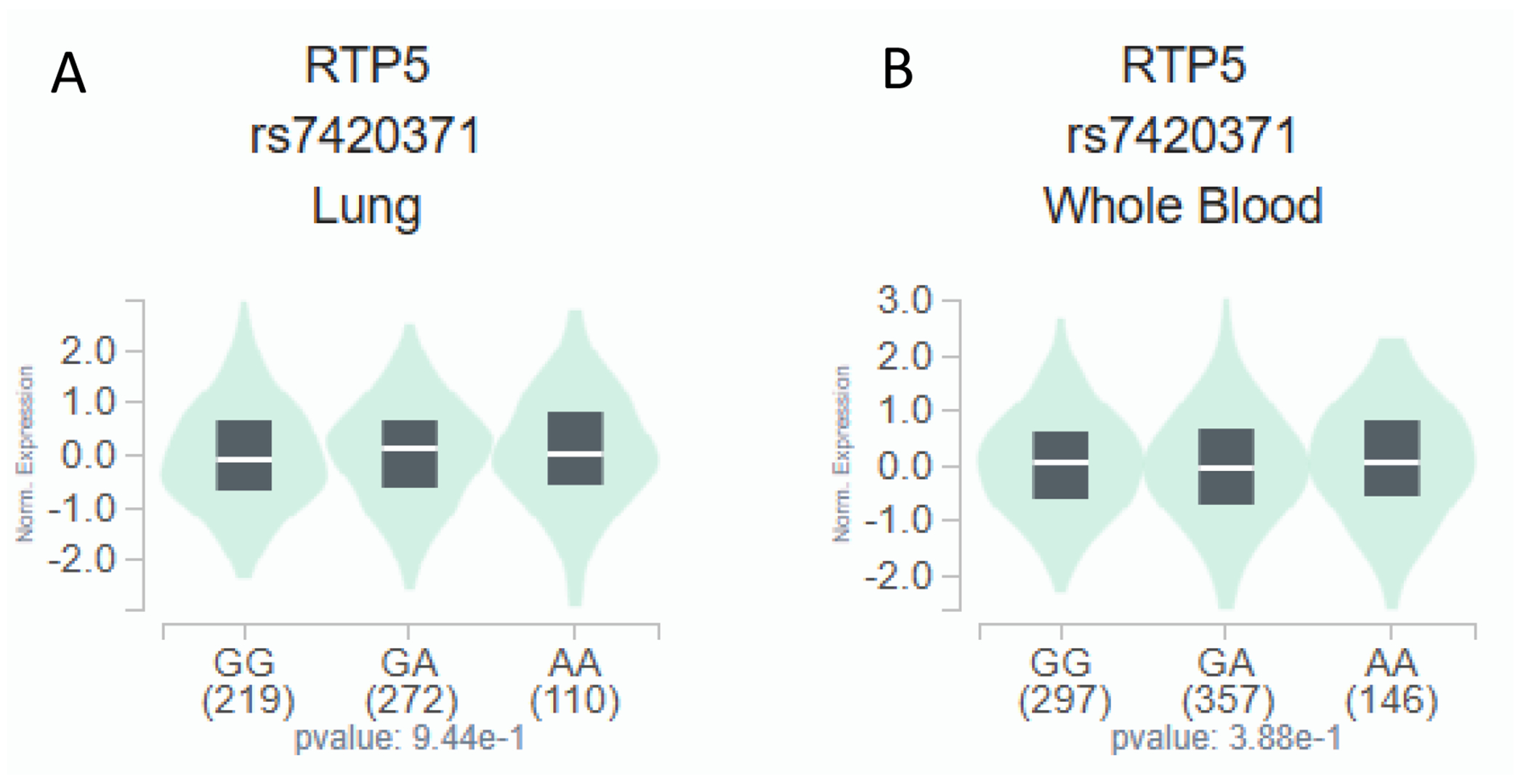
eQTL Analysis of rs7420371 G>A and mRNA Expression Levels of *RTP5* in the Lung and Whole Blood. The rs7420371 A allele is not statistically significantly associated with an increased expression of *RTP5* in 604 lung tissues from the GTEx database (A). The rs7420371 A allele is not statistically significantly associated with an increased expression of *RTP5* in 803 whole blood tissues from the GTEx database (B). Abbreviations: eQTL, expression quantitative trait loci; mRNA, messenger RNA; RTP5, receptor/chemosensory transporter protein 5.

**Table 1.** Clinical Characteristics of the 370 Severe COVID-19 Participants in the Genome-wide Association Study, Massachusetts General Hospital.

Covariates	Number of Patients (%)	30 Day Deaths (%)	60 Day Deaths (%)	Median Survival Time (Days) ^a^
Total	370	79 (21.4)	86 (23.2)	16.0

Age (Years)				
<50	86 (23.2)	8 (10.1)	9 (10.5)	16.5
≥50	284 (76.8)	71 (89.9)	77 (89.5)	16.0

Race & Ethnicity				
Non-Hispanic White	245 (66.2)	62 (78.5)	67 (77.9)	15.0
Hispanic	125 (33.8)	17 (21.5)	19 (22.1)	18.0

Sex				
Male	231 (62.4)	58 (73.4)	63 (73.3)	16.0
Female	139 (37.6)	21 (26.6)	23 (26.7)	15.0

Smoking Status				
Never	211 (57.0)	35 (44.3)	38 (44.2)	15.0
Former	127 (34.3)	31 (39.2)	35 (40.7)	17.0
Current	23 (6.2)	7 (8.9)	7 (8.1)	18.0

BMI (kg/m^2^)				
≤25	84 (22.7)	19 (24.1)	23 (26.7)	16.5
>25	286 (77.3)	60 (75.9)	63 (73.3)	15.5

ICU Status				
Floor	140 (37.8)	12 (15.2)	13 (15.1)	7.0
ICU	230 (62.2)	67 (84.8)	73 (84.9)	23.0

COVID Surge				
Wave 1 ^b^	203 (54.9)	40 (50.6)	41 (47.7)	19.0
Wave 2 ^c^	167 (45.1)	39 (49.4)	45 (52.3)	13.0

Abbreviations: BMI, body mass index; ICU, intensive care unit; MGH, Massachusetts General Hospital.

a Median survival time for time to death or discharge, whichever occurred first.

b Wave 1 patients are defined as adult ICU patients with SARS-CoV-2 infection and acute hypoxemic respiratory failure and floor (non-ICU) patients with mild hypoxemia managed with 2–6 L/min supplemental oxygen who were consecutively admitted to MGH between March-June 2020.

c Wave 2 patients are defined as adult ICU patients with SARS-CoV-2 infection and acute hypoxemic respiratory failure and floor (non-ICU) patients with mild hypoxemia managed with 2–6 L/min supplemental oxygen who were admitted to MGH between January–March 2021.

**Table 2.** Association of Top Genetic Signals with 30- and 60-Day Mortality in Severe COVID-19 Patients.

MGH COVID Genotypes (*n* = 370)									
Mortality	SNPs ^a^	Chromosome	Allele	MAF	Gene	FDR ^b^	Bonferroni ^c^	OR (95% CI) ^d^	SE
30-day	rs7420371	2	G>A	0.3926	*RTP5*	0.017	0.031	2.32 (1.59, 3.39)	0.19
60-day	rs7420371	2	G>A	0.3926	*RTP5*	0.027	0.050	2.06 (1.43, 2.97)	0.19

Abbreviations: MGH, Massachusetts General Hospital; SNPs, single nucleotide polymorphisms; FDR, false discovery rate; OR, odds ratio; SE, standard error.

a Remaining SNPs on respective chromosomes that are in high linkage disequilibrium (*r*^2^ > 0.80) are pruned and not shown.

b FDR threshold set at 0.05 under the Benjamini–Hochberg procedure. This is the FDR-adjusted *p*-value.

c Bonferroni threshold set at 0.05, assuming one million independent SNPs. This is the same threshold as the genome-wide significance level of 5 × 10^−8^.

d Adjusted for age, sex, smoking status, COVID surge, and the first four principal components.

**Table 3.** Optimized Sequence Kernel Association Test of Discovery Genome-wide Association Study ^a^ Signal and the COVID-19 Host Genetics Initiative.

Mortality Outcomes *p*-value		
Top GWAS Signal & HGI Signals	30-day Mortality ^b,c^	60-day Mortality ^b,c^
*RTP5* ^d^	5.90 × 10^−5^	6.17 × 10^−5^
*SFTPD*	2.04 × 10^−5^	3.85 × 10^−5^
*MUC5B*	1.42 × 10^−5^	3.46 × 10^−5^
*ELF5*	2.41 × 10^−6^	1.10 × 10^−5^
*FBRSL1*	1.76 × 10^−5^	2.23 × 10^−5^
*SLC22A31*	1.69 × 10^−5^	1.55 × 10^−5^
*TMPRSS2*	2.17 × 10^−5^	3.31 × 10^−5^
*NR1H2*	0.0001	0.0002
*THBS3*	0.0013	0.0006

Abbreviations: GWAS, genome-wide association study; HGI, COVID-19 host genetics initiative; SNPs, single nucleotide polymorphisms; FDR, false discovery rate.

a The discovery GWAS is the MGH cohort (*n* = 370).

b FDR threshold set at 0.05 under the Benjamini–Hochberg procedure. This is the FDR-adjusted *p*-value.

c Adjusted for age, gender, smoking status, COVID surge, and the top four principal components.

d Top independent SNP from GWAS based on *p*-value and selection after LD pruning.

## Data Availability

The data presented in this study are available on request from the corresponding author.

## Key Messages

### What is already known on this topic:

COVID-19 has been a leading cause of mortality in the United States for three consecutive years, with significant variability in disease severity among patients. Previous genetic association studies have identified several potential risk loci, but many findings lack replication or clear functional mechanisms.

### What this study adds:

The present study identifies a novel, independent genetic variant in the receptor transporter protein 5 gene (rs7420371 G>A) significantly associated with both 30-day and 60-day mortality in hospitalized COVID-19 patients. We provide supporting evidence that this variant influences *RTP5* gene expression in multiple tissues in the brain.

### How this study might affect research, practice or policy:

The identification of *RTP5*’s role in COVID-19 mortality provides hypothesis-generating insights into disease pathophysiology that may inform risk stratification approaches related to olfactory and taste receptor pathways disrupted by SARS-CoV-2 infection.
