# Supplementary material for: Gene-Level Analyses of Novel Olfactory-Related Signal from Severe SARS-CoV-2 GWAS Reveal Association with Disease Mortality
Source: COVID. Author manuscript; Available in PMC 2026 Mar 13. (PMC12981488; doi:10.3390/covid5120206)
Supplement: Table___Supplementary_Data [file NIHMS2147415-supplement-Table___Supplementary_Data.zip › Supplementary_Tables_YZ_v3.docx]

**Supplementary Table 1. Total Number of SNPs Retrieved and Matched for each Candidate Genetic Region.**

| **Top GWAS Signal & HGI Signals** | **Total Number of SNPs Retrieved^a^** | **Total Number of SNPs Matched^b^** |
| --- | --- | --- |
| *RTP5^c^* | 60,663 | 2,995 |
| *SFTPD* | 46,674 | 3,615 |
| *MUC5B* | 71,690 | 3,172 |
| *ELF5* | 50,664 | 3,102 |
| *FBRSL1* | 58,800 | 2,837 |
| *SLC22A31* | 78,557 | 3,108 |
| *TMPRSS2* | 54,183 | 3,093 |
| *NR1H2* | 6,3605 | 2,449 |
| *THBS3* | 53,701 | 1,099 |

Abbreviations: GWAS, genome-wide association study; HGI, COVID-19 host genetics initiative; SNPs, single nucleotide polymorphisms; GRCh37, genome reference consortium human build 37; QC, quality control; LD, linkage disequilibrium.

^a^Retrieved using the BiomaRt R package under the GRCh37 build with 500 kb flanking regions.

^b^Matched with the post-QC SNP list from the 370 severe COVID-19 patients. This is the number of SNPs considered in the SNP-set for each gene.

^c^Top independent SNP from GWAS based on *p*-value and selection after LD pruning, adjusted for age, sex, smoking status, covid surge, and the top four principal components.

**Supplementary Table 2. Functional Prediction of *RTP5* rs7420371 G>A using RegulomeDB and FAVOR.**

| RegulomeDB^a^ and FAVOR^e^ | | | | | | | | |
| --- | --- | --- | --- | --- | --- | --- | --- | --- |
| SNP | Gene | Chromosome | Score | ChIP-seq | Chromatin State | DNase | Selected eQTL hits | dbSNP Functional Annotation |
| rs74020371 | *RTP5* | 2 | 1f^b^ | NRF1^c^ | 833 hits | 29 tissues^d^ | 58 hits | Missense |
|  |  |  | aPC Protein Function | PHRED Percentile | PolyPhen Score | PolyPhen Classification | SIFT Score | SIFT Classification |
|  |  |  | 27.67 | 0.17 | 0.985 | Probably Damaging | 0 | Deleterious |

Abbreviations: FAVOR, functional annotation of variants online resource; SNP, single nucleotide polymorphism; ChIP-seq, chromatin immunoprecipitation sequencing; DNase, deoxyribonuclease; eQTL, expression quantitative trait loci; dbSNP, database for single nucleotide polymorphisms; PolyPhen, polymorphisms phenotyping; SIFT, sorting intolerant from tolerant.

^a^RegulomeDB: <http://regulomedb.org/>

^b^RegulomeDB score 1f is defined as eQTL evidence + TF binding or DNase peak.

^c^Target organ is bodily fluid and whole blood.

^d^29 tissues including the cardiac atrium, pancreas, and the lung.

^e^FAVOR: <https://favor.genohub.org/>

**Supplementary Table 3. Replication Attempts using Public Data from Release 7 of the COVID-19 Host Genetics Initiative**

| **Replication Cohort** | **Sample Size** | **OR** | **SE** | ***p*-value** |
| --- | --- | --- | --- | --- |
| Severe respiratory confirmed COVID-19 vs. Population Control | 18,152 cases; 1,145,546 controls | 0.99 | 0.014 | 0.5199 |
| Hospitalized vs. Non-hospitalized COVID-19 | 16,512 hospitalized cases; 71,321 non-hospitalized cases | 0.98 | 0.021 | 0.5203 |

Abbreviations: OR, odds ratio; SE, standard error.
