# Supplementary material for: Gene-Level Analyses of Novel Olfactory-Related Signal from Severe SARS-CoV-2 GWAS Reveal Association with Disease Mortality
Source: COVID. Author manuscript; Available in PMC 2026 Mar 13. (PMC12981488; doi:10.3390/covid5120206)
Supplement: Table___Supplementary_Data [file NIHMS2147415-supplement-Table___Supplementary_Data.zip › Tables_YZ_v3.docx]

**Table 1. Clinical Characteristics of the 370 Severe COVID-19 Participants in the Genome-wide Association Study, Massachusetts General Hospital**

| Covariates |  | Number of Patients (%) | | | 30 Day Deaths (%) | | 60 Day Deaths (%) | | Median Survival Time (Days)^a^ | |  |
| --- | --- | --- | --- | --- | --- | --- | --- | --- | --- | --- | --- |
| Total |  | 370 | | | 79 (21.4) | | 86 (23.2) | | 16.0 | |  |
| Age (Years) |  |  | | |  | |  | |  | |  |
| < 50  $\geq$ 50 | | | 86 (23.2) | 8 (10.1) | | 9 (10.5) | | 16.5 | |  |  |
|  |  |  | 284 (76.8) | 71 (89.9) | | 77 (89.5) | | 16.0 | |  |  |
| Race & Ethnicity | |  |  | | |  | |  | |  | |
| Non-Hispanic White  Hispanic | | | | 245 (66.2) | 62 (78.5) | | 67 (77.9) | | 15.0 | |  |
|  |  |  |  | 125 (33.8) | 17 (21.5) | | 19 (22.1) | | 18.0 | |  |
| Sex |  |  | | |  | |  | |  | |  |
| Male  Female | | | 231 (62.4) | 58 (73.4) | | 63 (73.3) | | 16.0 | |  |  |
|  |  |  | 139 (37.6) | 21 (26.6) | | 23 (26.7) | | 15.0 | |  |  |
|  |  |  |  |  | |  | |  | |  |  |
| Smoking Status |  |  | | |  | |  | |  | |  |
| Never  Former  Current | | | 211 (57.0) | 35 (44.3) | | 38 (44.2) | | 15.0 | |  |  |
|  |  |  | 127 (34.3) | 31 (39.2) | | 35 (40.7) | | 17.0 | |  |  |
|  |  |  | 23 (6.2) | 7 (8.9) | | 7 (8.1) | | 18.0 | |  |  |
|  |  |  |  |  | |  | |  | |  |  |
| BMI (kg/m^2^) |  |  | | |  | |  | |  | |  |
| $\leq$ 25  > 25 | | | 84 (22.7) | 19 (24.1) | | 23 (26.7) | | 16.5 | |  |  |
|  |  |  | 286 (77.3) | 60 (75.9) | | 63 (73.3) | | 15.5 | |  |  |
|  |  |  | | |  | |  | |  | |  |
| ICU Status |  |  | | |  | |  | |  | |  |
| Floor  ICU | | | 140 (37.8) | 12 (15.2) | | 13 (15.1) | | 7.0 | |  |  |
|  |  |  | 230 (62.2) | 67 (84.8) | | 73 (84.9) | | 23.0 | |  |  |
|  |  |  | | |  | |  | |  | |  |
| Covid Surge |  |  | | |  | |  | |  | |  |
| Wave 1^b^  Wave 2^c^ | | | 203 (54.9) | 40 (50.6) | | 41 (47.7) | | 19.0 | |  |  |
|  |  |  | 167 (45.1) | 39 (49.4) | | 45 (52.3) | | 13.0 | |  |  |

Abbreviations: BMI, body mass index; ICU, intensive care unit; MGH, Massachusetts General Hospital.

^a^Median survival time for time to death or discharge, whichever occurred first. .

^b^Wave 1 patients are defined as adult ICU patients with SARS-CoV-2 infection and acute hypoxemic respiratory failure and floor (non-ICU) patients with mild hypoxemia managed with 2-6 L/min supplemental oxygen who were consecutively admitted to MGH between March-June 2020.

^c^Wave 2 patients are defined as adult ICU patients with SARS-CoV-2 infection and acute hypoxemic respiratory failure and floor (non-ICU) patients with mild hypoxemia managed with 2-6 L/min supplemental oxygen who were admitted to MGH between January-March 2021.

**Table 2. Association of Top Genetic Signals with 30- and 60-Day Mortality in Severe Covid-19 Patients**

|  | | | |  | | **MGH COVID Genotypes (*n* = 370)** | | |  |  | |
| --- | --- | --- | --- | --- | --- | --- | --- | --- | --- | --- | --- |
| Mortality | SNPs^a^ | Chromosome | Allele | | MAF | | Gene | FDR^b^ | Bonferroni^c^ | OR (95% CI)^d^ | SE |
| 30-day | rs7420371 | 2 | G>A | | 0.3926 | | *RTP5* | 0.017 | 0.031 | 2.32 (1.59, 3.39) | 0.19 |
| 60-day | rs7420371 | 2 | G>A | | 0.3926 | | *RTP5* | 0.027 | 0.050 | 2.06 (1.43, 2.97) | 0.19 |

Abbreviations: MGH, Massachusetts General Hospital; SNPs, single nucleotide polymorphisms; FDR, false discovery rate; OR, odds ratio; SE, standard error.

^a^Remaining SNPs on respective chromosomes that are in high linkage disequilibrium (*r*^2^ >0.80) are pruned and not shown.

^b^FDR threshold set at 0.05 under the Benjamini-Hochberg procedure. This is the FDR-adjusted *p*-value.

^c^Bonferroni threshold set at 0.05, assuming one million independent SNPs. This is the same threshold as the genome-wide significance level of 5x10^-8^.

^d^Adjusted for age, sex, smoking status, covid surge, and the first four principal components.

**Table 3. Optimized Sequence Kernel Association Test of Discovery Genome-wide Association Study^a^ Signal and the COVID-19 Host Genetics Initiative.**

| **Mortality Outcomes P-value** | | |
| --- | --- | --- |
| **Top GWAS Signal & HGI Signals** | **30-day Mortality^b,c^** | **60-day Mortality^b,c^** |
| *RTP5^d^* | 5.90e-05 | 6.17e-05 |
| *SFTPD* | 2.04e-05 | 3.85e-05 |
| *MUC5B* | 1.42e-05 | 3.46e-05 |
| *ELF5* | 2.41e-06 | 1.10e-05 |
| *FBRSL1* | 1.76e-05 | 2.23e-05 |
| *SLC22A31* | 1.69e-05 | 1.55e-05 |
| *TMPRSS2* | 2.17e-05 | 3.31e-05 |
| *NR1H2* | 0.0001 | 0.0002 |
| *THBS3* | 0.0013 | 0.0006 |

Abbreviations: GWAS, genome-wide association study; HGI, COVID-19 host genetics initiative; SNPs, single nucleotide polymorphisms; FDR, false discovery rate.

^a^The discovery GWAS is the MGH cohort (n=370).

^b^FDR threshold set at 0.05 under the Benjamini-Hochberg procedure. This is the FDR-adjusted *p*-value.

^c^Adjusted for age, gender, smoking status, covid surge, and the top four principal components.

^d^Top independent SNP from GWAS based on *p*-value and selection after LD pruning.
