# Supplementary material for: Gene-Level Analyses of Novel Olfactory-Related Signal from Severe SARS-CoV-2 GWAS Reveal Association with Disease Mortality
Source: COVID. Author manuscript; Available in PMC 2026 Mar 13. (PMC12981488; doi:10.3390/covid5120206)
Supplement: Table___Supplementary_Data [file NIHMS2147415-supplement-Table___Supplementary_Data.zip › Supplementary_Figures_YZ_v3.docx]

**
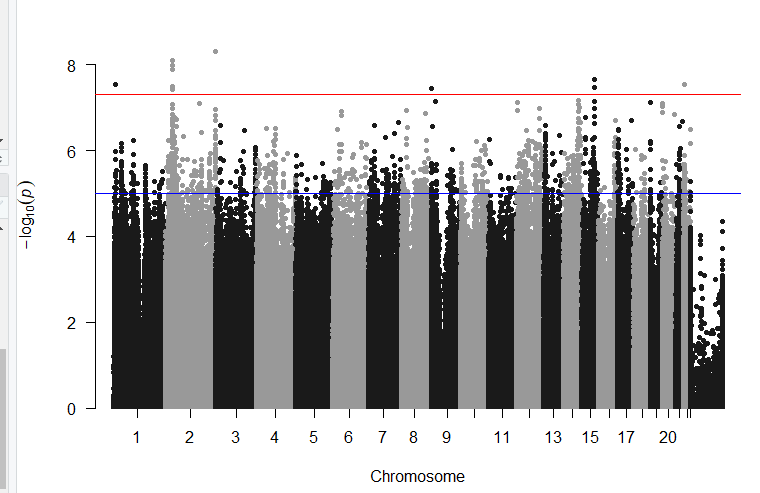
**

**rs7420371**

**Supplementary Figure 1A. Manhattan Plot for 30-Day Mortality**

**
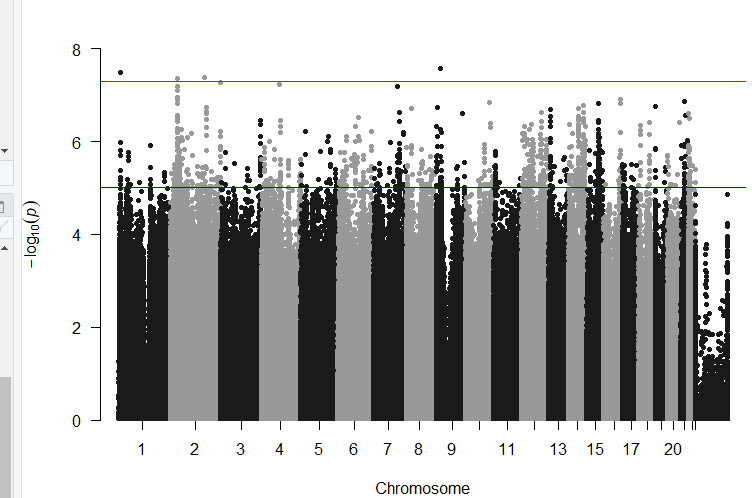
**

**rs7420371**

**Supplementary Figure 1B. Manhattan Plot for 60-Day Mortality**

**
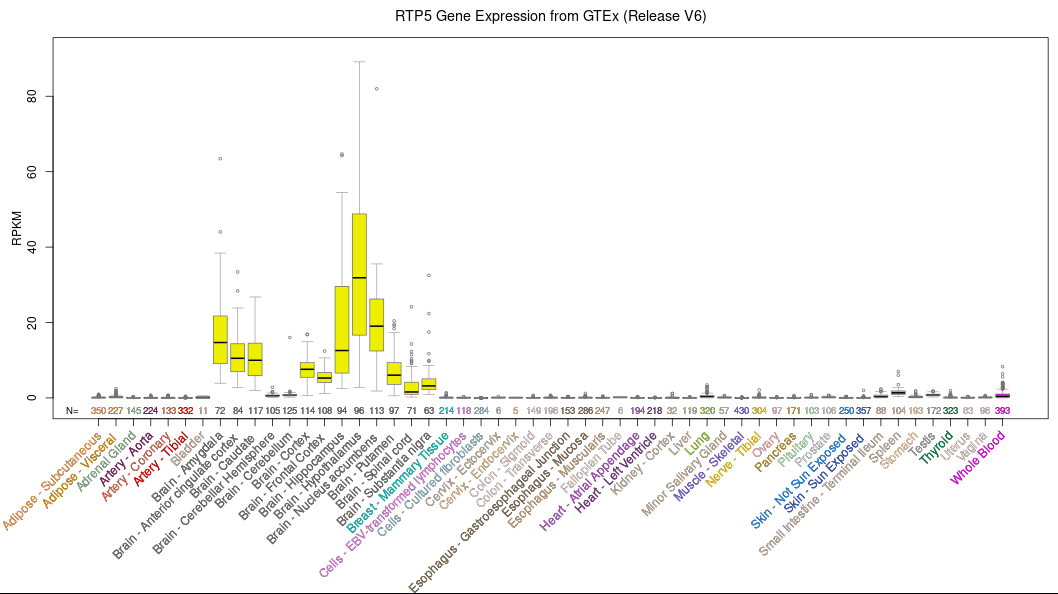
**

**Supplementary Figure 2. *RTP5* Expression Levels from GTEx.**

Abbreviations: RTP5, receptor/chemosensory transporter protein 5; GTEx, genotype-tissue expression portal; RPKM, reads per kilobase million.

The expression levels of *RTP5* by body site available from the GTEx project.


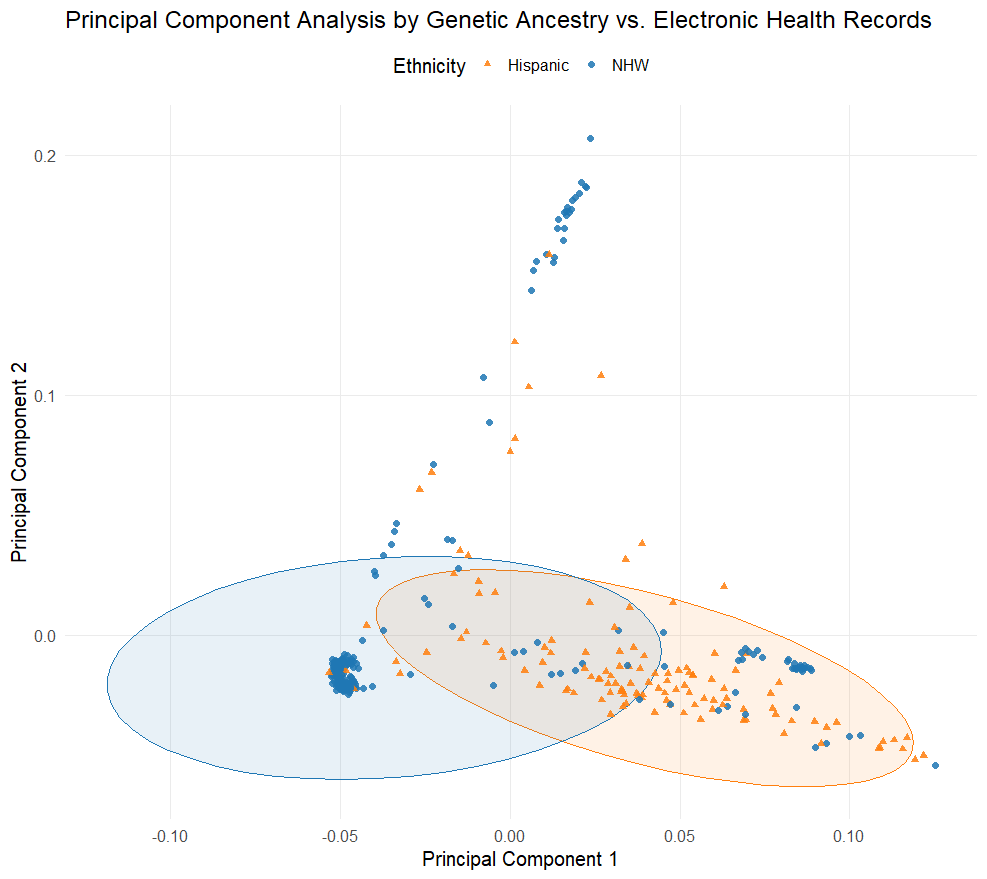


**Supplementary Figure 3. Principal Component Analysis by Genetic Ancestry vs Electronic Health Records.**

Abbreviations: NHW, non-Hispanic White; PCA, principal component analysis.
